# Teacher Adaptability and Student Development in Online Teaching Environments: A Survey of Teachers of Chinese Mathematics Competitions for Gifted Students

**DOI:** 10.3390/bs15050690

**Published:** 2025-05-17

**Authors:** Tianqi Lin, Peijie Jiang, Bin Xiong

**Affiliations:** 1 School of Mathematical Sciences, East China Normal University, Shanghai 200241, China; 52185500025@stu.ecnu.edu.cn; 2 Shanghai Key Laboratory of Pure Mathematics and Mathematical Practice, Shanghai 200241, China; 3 School of Mathematics and Statistics, Hunan Normal University, Changsha 410081, China

**Keywords:** online education, mathematics competition, teacher competencies, sustainable development, teacher adaptability, mediated moderation model

## Abstract

Despite advancements in information and technology, the benefits of online education for mathematically gifted students remain underexplored. In response, this study investigated the impacts of teacher competencies on students’ sustainable development in online mathematics competition education, examining the mediating role of teaching practice and the moderating role of teacher adaptability. Based on survey data from 289 Chinese mathematics competition teachers, the current research yielded the following findings: (1) Online teaching efficacy exerted a stronger positive influence on sustainable development compared with competition teaching professionalism, establishing the crucial role of technological competence in online education. (2) Teaching engagement and teaching practice significantly mediated the relationship between teacher competencies and sustainable development, with teaching engagement demonstrating stronger effects. (3) Teacher adaptability emerged as a significant moderator, empowering teaching practices and their effectiveness in promoting students’ sustainable development. These findings construct an integrated theoretical framework for understanding the translation of teacher competencies into student outcomes in online mathematics competition education, providing evidence-based guidance for enhancing teaching effectiveness in mathematics gifted education.

## 1. Introduction

### 1.1. Research Background

The rapid development of information technology is driving a profound digital transformation in education (Ministry of Education of China, 2024; Wang et al., 2024a; Zhou et al., 2025). This transformation aligns with United Nations Sustainable Development Goal 4 (SDG4), which emphasizes the promotion of quality education and lifelong learning opportunities for all (United Nations, n.d.; UNESCO, 2024). The spread of information technology has catalyzed the transformation of the global educational system, making educational resources more widely accessible while ensuring educational equity (UNESCO Institute for Information Technologies in Education, 2022; Wang et al., 2024a). Digital learning tools have substantially benefited higher education in a wide range of disciplines (e.g., in academic reading, see Lin & Yu, 2023). This democratization of education helps identify and develop mathematical talent that might otherwise remain dormant due to geographical, socioeconomic, or institutional barriers. By expanding access to high-quality educational resources, digital technologies create more chances to access quality educational resources and enable the discovery and nurturing of mathematically gifted students from diverse backgrounds, potentially broadening the pool of mathematical talent and thereby impacting national mathematical development (Tate & Warschauer, 2022; Xie et al., 2018).

In mathematics competition education, the digitalization of education is particularly significant. Mathematics competition students refer to those who participate in various levels of Mathematical Olympiad training and competitions. Those competitions have evolved through distinct stages in China since 1956, becoming an integral part of gifted education (Xiong & He, 2023). Through a multi-level selection mechanism, outstanding students ultimately represent China in the International Mathematical Olympiad (IMO). Mathematics competition participants often achieve remarkable academic and career success in their future development (Jung & Lee, 2021). As Kenderov (2022) noted, mathematics competitions have transformed from isolated events into a vibrant global ecosystem that significantly impacts talent identification and development. The advancements of modern technologies have brought mathematics competition education into a new era of technological integration in recent years.

#### Online Mathematics Competition Education in China

In Chinese contexts, students participating in mathematics competition training often comprise a crucial proportion of the mathematically gifted student population, demonstrating exceptional mathematical talent, solid foundations, and strong problem-solving abilities. Research indicates that excellent students maintain engagement and motivation when facing challenging tasks aligned with their interests and abilities (Procopio et al., 2022).

Online mathematics competition education in China differs from both traditional face-to-face formats and regular mathematics education in several significant ways. Unlike regular mathematics education, which follows the national curriculum with standardized content and pacing, mathematics competition education emphasizes advanced problem-solving, creative thinking, and mathematical exploration beyond the standard curriculum (Xiong & He, 2023). This specialized education typically targets students identified as mathematically gifted, who demonstrate exceptional mathematical aptitude and interest.

The transition to online formats has further differentiated this educational approach. While face-to-face mathematics competition education allows for immediate feedback, physical demonstrations, and close mentorship, the online mode can substantially re-create these dynamics through digital tools and pedagogical innovations (Xu et al., 2024). This shift introduces unique challenges for teachers, who must learn to efficiently use educational technologies and adapt their instructional strategies and resources to maintain student engagement and facilitate collaborative problem-solving in virtual environments.

The differences between online mathematics competition education and regular online mathematics instruction are also substantial. Regular online mathematics courses typically focus on helping students master standard curriculum content, while competition-oriented education emphasizes developing advanced mathematical thinking, creative problem-solving strategies, and preparation for high-level competitions (Kenderov, 2022). These differences necessitate specialized teaching approaches and competencies that are the focus of the current study.

### 1.2. Problem Statement and Motivations of Current Study

While previous research has explored the relationship between online and mathematics competition education (Xu et al., 2024), several critical questions remain unclear regarding the mechanism between teachers’ online teaching competencies and gifted students’ sustainable development in mathematics competitions. Online gifted education requires teachers to have unique adaptability and innovative capabilities. The role of teachers in this context is paramount, as they are responsible for nurturing the mathematical abilities of gifted students. In contrast to a large body of empirical studies from students’ perspectives, teachers’ perceptions and competencies have yet to be widely explored. Additionally, Lee (2023) highlighted specific difficulties that newly employed teachers face in gifted science and mathematics education, particularly in determining the appropriate levels of enriched content and considering the characteristics of heterogeneous groups. The role of their adaptability has yet to be extensively explored in this specific context, though it may help to avoid the negative impacts of the challenges of the digital shift toward innovative teaching and learning modes.

Thus, the current study was motivated by the following research gaps. First, despite online education offering new opportunities for teaching innovation (Johnson et al., 2021), the effective translation of traditional gifted education theories into online educational environments remains unclear. Second, while research has examined teachers’ online teaching efficacy (Frazer & Sullivan, 2017), studies combining this with mathematics competition education are limited. Currently, there are a lack of theoretical models explaining the sustainability of online mathematics competition education.

### 1.3. Research Objectives

Based on the research gaps, the current study has three objectives, as follows:

1. To investigate the impact of teacher competencies on students’ sustainable development according to teachers’ ratings, i.e., how competition teaching professionalism, online teaching efficacy, and the understanding of gifted students influence students’ teacher-rated academic achievement, creative thinking, and problem-solving abilities.
2. To analyze the mediating role of the teaching process, i.e., how teaching engagement, practical activities, and feedback mechanisms mediate the relationship between teacher competencies and students’ sustainable development.
3. To examine the moderating role of teacher adaptability in the relationship between teacher competencies and students’ sustainable development.

## 2. Literature Review and Theoretical Framework

### 2.1. Professional Characteristics of Mathematics Competition Education

Mathematics competition education places unique demands on teachers’ professional competencies, especially in developing gifted students’ mathematical creativity and problem-solving abilities. According to Kenderov (2022), mathematics competitions evolved from isolated events into a comprehensive educational ecosystem requiring specialized teaching approaches. Through large-scale empirical research, Hill et al. (2005) confirmed that teachers’ Mathematical Knowledge for Teaching levels significantly correlated with students’ mathematical achievement. Motivated by earlier studies, an updated Mathematical Knowledge for Teaching (MKT) framework was proposed by Ball et al. (2008) to provide a systematic perspective for understanding teachers’ professional knowledge, clearly defining the specific content and pedagogical knowledge required for teachers’ successful educational practice. In competitive mathematics education, teachers need the ability to design and implement multiple-solution teaching, which directly affects students’ creative thinking development (Leikin, 2011). Additionally, other evidence has suggested teachers’ crucial role in cultivating students’ mathematical creativity and problem-solving abilities (Sriraman, 2008).

In mathematics competition education, sustainable developments extend beyond immediate academic performance. As Geraniou and Jankvist (2019) argue, it encompasses the development of students’ long-term mathematical thinking, problem-solving capabilities, and the integration of both traditional mathematical competencies and digital competencies. This comprehensive view of sustainable development aligns with VanTassel-Baska and Stambaugh’s (2009) emphasis on long-term talent development and Gagné’s (2015) DMGT for talent development. More specifically, Gagné’s model combined two classic models, i.e., the “Differentiated Model of Giftedness and Talent” and the “Developmental Model for Natural Abilities”. Gagné’s (2015) model examined talent development as the formation of excellent particular knowledge and skills from natural abilities, which could be catalyzed by intrapersonal and environmental factors. This integrated model clarified how talent education could be improved by considering more contextual and situational factors.

### 2.2. Technology-Supported Teaching Integration

Online educational environments brought challenges to mathematics competition education, particularly for gifted mathematics students. According to Xu et al. (2024), online education significantly impacts gifted mathematics students’ learning patterns and achievements, with different effects across various student groups. The intersection of pedagogical practice and technological integration was established early on by the Technological, Pedagogical, and Content Knowledge (TPACK) framework proposed by Mishra and Koehler (2006), which systematically explained the dynamic integration relationship between technology, pedagogy, and content knowledge, providing a theoretical foundation for understanding online teaching. This framework emphasized that effective technology integration required teachers to understand the complex interactions between technology, pedagogy, and content knowledge.

Through systematic research, Borba et al. (2016) found that digital technology applications created new learning opportunities in mathematics education, but that this required teachers to develop corresponding teaching strategies. Drijvers et al. (2016) further point out that effective technology application in mathematics education requires teachers to have specific professional competencies and instructional design skills. However, criticisms claimed that TPACK alone provided “a rather coarse-grained tool” for analyzing teachers’ knowledge and therefore required complementing with other frameworks to achieve an adequate depth of analysis (Ruthven et al., 2004). This was particularly relevant in online mathematics competition education, where teachers needed to integrate specialized mathematical content knowledge with technological pedagogical skills.

Furthermore, recent research by Geraniou and Jankvist (2019) emphasized that, in modern mathematics classrooms, teachers’ technological and pedagogical competencies were deeply intertwined. Their work suggested that effective mathematics teaching in digital environments required teachers to develop what they termed “mathematical digital competencies”. This concept provided a crucial theoretical foundation for understanding how online teaching efficacy impacted teaching engagement and practice. Additionally, Drijvers et al. (2016) argued that effective technology integration required teachers to be highly engaged in orchestrating digital learning environments through what Trouche (2004) termed “instrumental orchestration” to reflect the teacher’s intentional and systematic organization and use of various digital artifacts in mathematical learning environments.

### 2.3. Teacher Adaptability Theory

#### 2.3.1. Conceptualization and Significance

Teacher adaptability plays a crucial role in online educational environments, particularly in gifted education. (Ronksley-Pavia & Neumann, 2022) emphasize that online gifted education requires teachers to have unique adaptability and innovative capabilities in order to effectively engage gifted students. The teacher adaptability theoretical framework was proposed by Collie and Martin (2016). They believed that the tripartite model of cognition, emotion, and behavior could be effectively applied in research on primary and secondary schools, as well as university students. Through empirical research, König et al. (2020) confirmed that teacher adaptability significantly impacted teaching effectiveness in online teaching environments, particularly in maintaining teaching quality when facing sudden changes in the teaching environment. Their research further revealed that teacher adaptability significantly moderated the relationship between teaching practices and learning outcomes in online teaching environments. This finding highlighted how teachers’ adaptive capabilities could enhance both the implementation of teaching strategies and the effectiveness of student learning outcomes.

#### 2.3.2. Multidimensional Nature of Teacher Adaptability

Teacher adaptability in online mathematics education should be conceptualized as a multidimensional construct encompassing cognitive, emotional, and behavioral aspects (Collie & Martin, 2016). The cognitive dimension involves teachers’ capacity to adjust their thinking and instructional approaches when faced with novel teaching situations. This includes recognizing when existing strategies are ineffective and generating alternative approaches tailored to the online environment (Baran et al., 2011).

The emotional dimension of adaptability refers to teachers’ ability to regulate their affective responses to challenging situations, maintaining positive engagement despite technological disruptions or student learning difficulties (Taxer & Gross, 2018). This emotional resilience is particularly crucial in online mathematics competition education, where students’ frustration with challenging problems can be more difficult to detect and address remotely.

The behavioral dimension encompasses teachers’ capacity to modify their instructional practices in response to changing circumstances (König et al., 2020). In online mathematics competition education, this manifests as the flexible implementation of teaching strategies, creative use of digital tools, and responsive modification of task difficulty based on student performance.

These dimensions interact dynamically, with cognitive adaptability informing behavioral responses, emotional adaptability supporting persistent engagement, and behavioral adaptability providing feedback that reshapes cognitive understanding. Recent empirical research has demonstrated that these adaptability dimensions predict teacher effectiveness in online environments more strongly than traditional measures of teaching competence (MacMahon et al., 2022).

### 2.4. Mediating Mechanisms of Teaching Process

Teachers’ professional competencies are transformed into teaching effects through the teaching process, particularly in online gifted education contexts. Lee (2023) highlighted the specific challenges that teachers faced in science and mathematics gifted education, emphasizing the importance of appropriate content levels and the consideration of heterogeneous group characteristics. Ball et al.’s (2008) research showed that teachers’ mathematical teaching knowledge needed to be facilitated through effective teaching practices in order to promote student development. Their study echoed Hill et al. (2005), who confirmed a significant association between teachers’ professional knowledge and student learning outcomes, realized through the teaching process. The transformation of teacher competencies into teaching effects requires careful orchestration in online environments.

### 2.5. Toward an Integrated Framework of Online Mathematics Competition Education

Recent research has highlighted several emerging trends in online gifted mathematics education. Xu et al. (2024) found that online education had different impacts on gifted mathematics students from various family backgrounds, suggesting the need for differentiated teaching approaches. (Ronksley-Pavia & Neumann, 2022) emphasized the importance of educator leadership practices in online gifted education, particularly in maintaining student engagement. With the increasingly pronounced advantages, popularity, and complexity of online mathematics competition education, it is imperative for researchers and teachers to synthesize theoretical bases from the previous literature.

#### 2.5.1. Integration of Theoretical Frameworks

For the current study, we synthesized Mathematical Knowledge for Teaching (Ball et al., 2008), TPACK (Mishra & Koehler, 2006), and teacher adaptability (Collie & Martin, 2016; Collie & Hascher, 2024). The above theoretical bases reflect differentiated emphases in teaching practice. The MKT framework emphasizes how teachers’ specialized content knowledge informs their pedagogical decisions and classroom interactions. The TPACK framework extends this by examining how technological knowledge integrates with pedagogical and content knowledge to shape teaching practice in digital environments. The teacher adaptability framework further illuminates how teachers’ capacity to adjust their cognitive, emotional, and behavioral responses moderates the effectiveness of these teaching processes.

However, these theories could be made complementary to each other in order to form more complete guidelines for teachers in digital educational contexts. The theoretical frameworks above converge around a central focus on teaching processes. These processes serve as the crucial mechanisms through which teacher competencies can be translated into student outcomes. Together, these frameworks provide a comprehensive lens for understanding how teacher competencies influence teaching processes, which in turn affect student outcomes. This integrated perspective is particularly valuable in the context of online mathematics competition education, where teaching processes must simultaneously address advanced mathematical content, leverage digital tools effectively, and adapt to the unique needs of gifted students.

#### 2.5.2. Integration of MKT and TPACK Frameworks

The relationship between the Mathematical Knowledge for Teaching (MKT) framework (Ball et al., 2008) and the Technological, Pedagogical, and Content Knowledge (TPACK) framework (Mishra & Koehler, 2006) represents an important theoretical integration in our study. Several researchers have explored the intersection of these frameworks, particularly in technology-enhanced mathematics education (Niess et al., 2009; Bowers & Stephens, 2011).

MKT provides a detailed conceptualization of the specialized knowledge required for effective mathematics teaching, including common content knowledge, specialized content knowledge, knowledge of content and students, and knowledge of content and teaching. This framework offers deep insights into the mathematical aspects of teacher competence but does not specifically address the technological dimensions of teaching.

TPACK extends this perspective by explicitly incorporating technological knowledge and examining how it interacts with pedagogical and content knowledge. In mathematics education specifically, TPACK addresses how teachers integrate digital tools to represent mathematical concepts, facilitate problem-solving, and create innovative learning experiences (Niess, 2005).

In our theoretical model, competition teaching professionalism (CTP) draws heavily from the MKT framework, focusing on teachers’ specialized mathematical knowledge in competition contexts. Online teaching efficacy (OTE), meanwhile, incorporates elements of the TPACK framework, emphasizing teachers’ ability to effectively integrate technology into mathematics instruction. By including both constructs in our model, we acknowledge both the depth of mathematical knowledge required (MKT) and the technological integration skills needed (TPACK) in online mathematics competition education.

This integration is particularly relevant for hypothesis H1a, which proposes that competition teaching professionalism positively impacts sustainable development. While our rationale primarily references MKT-related research, the implementation of this professional knowledge in online environments inherently involves technological integration as conceptualized in the TPACK framework. Future research could further explore the specific interactions between MKT and TPACK elements in online mathematics competition teaching.

#### 2.5.3. Significance for Synthesizing Theoretical Frameworks

Combining the theoretical backgrounds above may help to explain several key phenomena. First, drawing on Ball et al.’s (2008) MKT theory and Mishra and Koehler’s (2006) TPACK framework, researchers can better understand how teachers transform their professional knowledge in technology-mediated environments. Second, following König et al.’s (2020) research on teacher adaptability in online environments, we can explain why some teachers maintain effectiveness during technological transitions while others struggle.

The practical applications of integrating these theoretical frameworks extend to teachers’ professional development. As highlighted by Lee (2023), teachers in mathematically gifted education faced specific challenges in determining appropriate content levels and considering the characteristics of heterogeneous groups. An integrated framework may suggest that effective professional development should simultaneously address content knowledge (Ball et al., 2008), technological integration (Drijvers et al., 2016), and adaptive capabilities (Collie & Martin, 2016).

This theoretical integration can also provide insights into future research directions. As (Ruthven, 2014) suggested, researchers would need a “fuller and more systematic investigation of the phenomenon of technology integration into subject teaching”. Our perspective of an integrated theoretical framework provides a foundation for such an investigation, particularly in the context of online mathematics competition education.

These findings suggest that successful online mathematics competition education requires a comprehensive understanding of both technological and pedagogical aspects, as well as the consideration of student diversity and engagement factors. The integration of these theoretical perspectives provides a more nuanced understanding of how different aspects of teacher competencies interact to influence student outcomes in online educational environments.

## 3. Research Hypotheses and Theoretical Model Construction

### 3.1. Conceptual Model Overview

**Figure 1:**
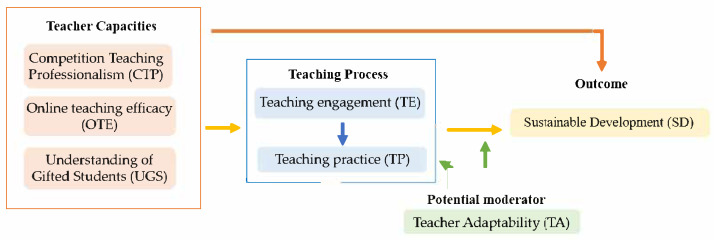
The integrated theoretical framework of the current study. Note. CTP: Competition teaching professionalism. OTE: Online teaching efficacy. UGS: Understanding of gifted students. TE: Teaching engagement. TP: Teaching practice. SD: Sustainable development. TA: Teacher adaptability.

Based on the aforementioned theoretical frameworks, this study constructs a comprehensive research model integrating exogenous, mediating, moderating, and outcome variables (Figure 1). The conceptual model includes the following key variables: competition teaching professionalism (ξ1), online teaching efficacy (ξ2), and the understanding of gifted students (ξ3) function as exogenous latent variables, reflecting the core dimensions of teacher competencies; teaching engagement (η1) and teaching practice (η2) function as mediating latent variables, embodying the process mechanisms of transforming teacher competencies into teaching effectiveness; teacher adaptability (ξ4) functions as a moderating variable, and we examine its influence on the teaching process; and sustainable development (η3) functions as the dependent variable, measuring the ultimate educational effect. The subsequent sections will elaborate on how our research hypotheses were developed based on the interrelationships of those elements.

### 3.2. Research Hypotheses

#### 3.2.1. Direct Effect Hypotheses

According to the Mathematical Knowledge for Teaching (MKT) framework, teachers’ professional knowledge systematically affects teaching quality and student development. Through extensive empirical research, Hill et al. (2005) confirmed that teachers’ MKT levels correlated with students’ mathematical achievement. In competitive mathematics education in particular, Leikin (2011) found that teachers’ professional knowledge was crucial for developing students’ creative thinking and problem-solving abilities.

Researchers have established the critical role of teacher professionalism in gifted education. Opoku et al. (2023) demonstrated that teacher professional competence directly impacted gifted students’ learning outcomes. Additionally, Lee (2023) found that mathematics competition teachers’ professional knowledge significantly influenced students’ problem-solving abilities and creative thinking. In online environments, Li (2024) confirmed that teachers’ professional capabilities remained crucial for maintaining teaching effectiveness. However, for an overall outcome variable of students’ sustainable development, the positive effect of teacher professionalism in gifted education remained unclear in our research context, which led us to the following hypothesis:

**H1a.**  *Competition teaching professionalism (CTP) has a positive impact on sustainable development (SD).*

In online educational environments, Mishra and Koehler’s (2006) TPACK framework systematically explains the dynamic integration of technology, pedagogy, and content knowledge. Rich and comprehensive knowledge related to online teaching should allow teachers to develop their teaching efficacy in innovative contexts. Borba et al. (2016) found that digital technology applications created new learning opportunities in mathematics education. Drijvers et al. (2016) further emphasized that effective technology application required teachers to have specific professional competencies. It is presumable that quality online teaching practice supported by relevant knowledge and high teacher efficacy should result in better learning outcomes. Based on these findings, the following hypothesis (H1b) is proposed to validate the effect in our research context:

**H1b.**  *Online teaching efficacy (OTE) has a positive impact on sustainable development (SD).*

Understanding student characteristics was a premise for quality teaching, and hence students’ sustainable development in mathematics competition education. VanTassel-Baska and Stambaugh’s (2009) research showed that teachers’ understanding of gifted students’ characteristics significantly predicted students’ long-term learning outcomes. According to Gagné’s (2015) DMGT, teachers were among the environmental catalysts that directly influenced students’ talent development, a process Gagné viewed as a transfer from abilities to knowledge and skills. Geraniou and Jankvist (2019) argued that, in online environments specifically, effective mathematics teaching requires teachers to develop a deep understanding of how gifted students learn in digital contexts. This integrated understanding suggested the following hypothetical link (H1c):

**H1c.**  *The understanding of gifted students (UGS) has a positive impact on sustainable development (SD).*

#### 3.2.2. Teaching Engagement Effect Hypotheses

Based on Hill et al.’s (2005) teacher knowledge transformation model and recent findings from S. Huang et al. (2022) regarding the predictive role of self-efficacy in engagement, teachers’ professional knowledge needs to be transformed through teaching engagement. Dong et al. (2023) found that teacher adaptability significantly moderated changes in online teaching self-efficacy. Thus, the following hypothesis was proposed:

**H2a.**  *Competition teaching professionalism (CTP) has a positive impact on teaching engagement (TE).*

In technology-supported teaching environments, Mishra and Koehler (2006) found that teachers’ technological pedagogical knowledge significantly influenced their teaching engagement. To contribute to this theoretical stance, we proposed the following hypothesis:

**H2b.**  *Online teaching efficacy (OTE) has a positive impact on teaching engagement (TE).*

Subotnik et al.’s (2011) research demonstrated that teachers’ understanding of gifted students significantly enhanced their teaching engagement. According to Ball et al. (2008), teaching engagement directly influenced teaching practice quality. Hattie’s (2009) meta-analysis confirmed that teaching engagement affected student development. To test these relationships in our research context, we proposed the following research hypotheses:

**H2c.**  *The understanding of gifted students (UGS) has a positive impact on teaching engagement (TE).*

**H2d.**  *Teaching engagement (TE) has a positive impact on teaching practice (TP).*

**H2e.**  *Teaching engagement (TE) has a positive impact on sustainable development (SD).*

#### 3.2.3. Teaching Practice Effect Hypotheses

Ball et al.’s (2008) research indicated that teachers’ professional knowledge needed to be implemented through effective teaching practices. Drijvers et al. (2016) emphasized that technological integration required appropriate instructional design and implementation. VanTassel-Baska & Stambaugh’s (2009) research showed that the understanding of gifted students needed to be transformed into effective educational support. To establish the role of those three elements, we proposed the following research hypotheses:

**H3a.**  *Competition teaching professionalism (CTP) has a positive impact on teaching practice (TP).*

**H3b.**  *Online teaching efficacy (OTE) has a positive impact on teaching practice (TP).*

**H3c.**  *The understanding of gifted students (UGS) has a positive impact on teaching practice (TP).*

#### 3.2.4. Sequential Mediation Effect Hypotheses

Among the proposed elements, chain effects might be explored to elucidate their interrelatedness and detailed mechanisms. Based on Hill et al.’s (2005) model and Hattie’s (2009) meta-analysis findings, the following hypothesis was proposed:

**H4a.**  *Teaching engagement (TE) and teaching practice (TP) sequentially mediate the relationship between competition teaching professionalism (CTP) and sustainable development (SD).*

In technology-supported environments, several theoretical perspectives support this mediation relationship. Mishra and Koehler’s (2006) TPACK framework emphasizes the integration of technological and pedagogical knowledge. According to Trouche’s (2004) instrumental orchestration theory, teachers’ technological efficacy must be transformed through their active engagement and through systematic teaching practices to impact student outcomes. Furthermore, Drijvers et al. (2016) emphasize that effective technology integration in mathematics education requires specific instructional design skills developed through sustained teaching engagement. This theoretical integration suggested the following hypothesis for us to test:

**H4b.**  *Teaching engagement (TE) and teaching practice (TP) sequentially mediate the relationship between online teaching efficacy (OTE) and sustainable development (SD).*

Subotnik et al.’s (2011) research on gifted education motivated us to develop the following hypothesis:

**H4c.**  *Teaching engagement (TE) and teaching practice (TP) sequentially mediate the relationship between the understanding of gifted students (UGS) and sustainable development (SD).*

#### 3.2.5. Simple Mediation Effect Hypotheses

Drawing on multiple theoretical perspectives, including Hattie’s (2009) meta-analysis on teaching effectiveness, Drijvers et al.’s (2016) work on technology integration, and Trouche’s (2004) instrumental orchestration theory, we proposed that teacher competencies might influence student outcomes through both direct and indirect pathways. As Geraniou and Jankvist (2019) argue, these relationships are particularly complex in digital mathematics learning environments. Therefore, the following hypotheses were proposed:

**H5a.**  *Teaching engagement (TE) mediates the relationship between competition teaching professionalism (CTP) and sustainable development (SD).*

**H5b.**  *Teaching practice (TP) mediates the relationship between competition teaching professionalism (CTP) and sustainable development (SD).*

**H5c.**  *Teaching engagement (TE) mediates the relationship between online teaching efficacy (OTE) and sustainable development (SD).*

#### 3.2.6. Moderation Effect Hypotheses

The dual moderating role of teacher adaptability can be theoretically explained through the integration of multiple frameworks. Ruthven et al. (2004) points out that technology integration requires teachers to continuously adapt their engagement and practices to the digital environment. The combination of these perspectives suggested the following hypotheses for the current study:

**H6a.**  *Teacher adaptability (TA) moderates the relationship between teaching engagement (TE) and teaching practice (TP).*

**H6b.**  *Teacher adaptability (TA) moderates the relationship between teaching practice (TP) and sustainable development (SD).*

### 3.3. Innovative Value of Theoretical Model

The theoretical model constructed in this study contains the following innovative characteristics:

First, it integrates multiple dimensions of teacher competencies. Unlike previous studies’ singular examination of teacher capabilities, this model incorporates competition teaching professionalism (CTP), online teaching efficacy (OTE), and the understanding of gifted students (UGS) into a unified analytical framework, more comprehensively reflecting the composition of teacher competencies in online mathematics competition education.

Second, a complex intermediary mechanism is constructed. By introducing two mediating variables, teaching engagement (TE) and teaching practice, and by examining their simple mediating effects and sequential mediating effects, the mechanism by which teacher ability affects student development is revealed in depth.

Third, the moderating effect of teacher adaptability (TA) is introduced. This innovative design not only reflects the important impact of teachers’ adaptability on teaching effectiveness but also provides new ideas for improving teaching quality.

### 3.4. Practical Significance of Theoretical Model

The theoretical model in this research has significant practical implications for online mathematics competition education:

First, it provides a clear direction for teachers’ professional development. The model emphasizes the cultivation of key competencies, including competition teaching professionalism (CTP), online teaching efficacy (OTE), and the understanding of gifted students (UGS), indicating specific pathways for enhancing teacher capabilities.

Second, it offers theoretical guidance for improving teaching processes. By revealing the mediating roles of teaching engagement (TE) and teaching practice in transforming teacher competencies into educational outcomes, the model provides concrete approaches for improving teaching processes.

Third, it highlights the crucial value of teacher adaptability. By examining the moderating role of teacher adaptability, the model presents new focal points for improving educational quality, which is particularly significant in online teaching environments.

## 4. Research Methods

### 4.1. Research Design and Data Collection

#### 4.1.1. Survey Development

This research employed the “Online Mathematics Competition Teacher Survey” as its primary data collection instrument. The questionnaire (Appendix A) consisted of two main sections:

(1) Demographic information: This section collected data regarding teachers’ (a) gender, (b) age, (c) years of teaching experience, (d) competition teaching experience, and (e) school type.

(2) Core construct measurement: The survey included 28 measurement items using a 7-point Likert scale (1 = “strongly disagree” to 7 = “strongly agree”). These items measured competition teaching professionalism (CTP), online teaching efficacy (OTE), the understanding of gifted students (UGS), teaching engagement (TE), teaching practice (TP), sustainable development (SD), and teacher adaptability (TA).

#### 4.1.2. Measurement Tool Development

Based on established theoretical frameworks and the literature, the following measurement scales were developed, with 28 items in total:

**Competition teaching professionalism scale (CTP, ξ1)**: Drawing on Ball et al.’s (2008) MKT theory, this scale used four items (CTP1-CTP4) to assess teachers’ professional capabilities in mathematics competition teaching, including knowledge systems, problem-solving strategies, mathematical thinking cultivation, and competition pattern understanding.

**Online teaching efficacy scale (OTE, ξ2)**: Based on Tschannen-Moran and Hoy’s (2001) TSES framework, this scale employed four items (OTE1–OTE4) to evaluate teachers’ proficiency in online platform usage, course design, resource integration, and technical problem-solving.

**Understanding of gifted students scale (UGS, ξ3)**: This four-item scale (UGS1–UGS4) measured teachers’ understanding of gifted students’ learning. It was created in-house according to the essential components of this concept, including learning characteristics, needs, guidance requirements, and psychological traits.

**Teaching engagement scale (TE, η1)**: Adapted from Schaufeli et al.’s (2002) UWES scale, this scale used four items (TE1-TE4) to assess teachers’ time investment, reflection, participation, and professional development in teaching.

**Teaching practice scale (TP, η2)**: This in-house-developed scale employed four items (TP1-TP4) to evaluate teachers’ goal setting, online classroom organization, teacher–student interactions, and teaching strategy adjustment. The scale was developed specifically for this study based on the theoretical frameworks of Ball et al. (2008) and Drijvers et al. (2016), and was validated through expert review and pilot testing.

**Sustainable development scale (SD, η3)**: This scale used four items (SD1-SD4) to measure students’ development outcomes as perceived by teachers. Specifically, SD1 assessed competition performance (“My students have shown improved performance in mathematics competitions”), SD2 measured problem-solving ability (“My students have demonstrated enhanced mathematical problem-solving abilities”), SD3 evaluated innovative thinking (“My students have developed more creative approaches to mathematical problems”), and SD4 assessed long-term learning motivation (“My students maintain strong interest and motivation in mathematical learning”). This scale was developed based on VanTassel-Baska and Stambaugh’s (2009) work on talent development and Gagné’s (2015) DMGT and adapted for the Chinese mathematics competition context.

**Teacher adaptability scale (TA, ξ4)**: This scale was adapted from Collie and Martin’s (2016) teacher adaptability measure and modified for the online mathematics competition education context. The scale used four items (TA1–TA4) to assess teachers’ ability to adapt to new environments, innovate teaching methods, adjust teaching plans, and respond to challenges.

#### 4.1.3. Data Collection

The study collected data through an online survey platform, targeting mathematics competition teachers across multiple regions in China. A pilot test was conducted to ensure item clarity and comprehensibility. The final survey yielded 289 valid responses. The dataset formed a sufficient and valid sample, following previous studies’ practice and suggestions on using Structural Equation Modeling (PLS-SEM) in educational research (e.g., Lin et al., 2023; Wang et al., 2023).

The study employed a convenience sampling method rather than random selection, targeting mathematics competition teachers who were accessible through professional networks and educational institutions. This sampling method has been widely applied in educational research since it allows researchers to make the least amount of effort in finding eligible samples related to the current research (e.g., Lin & Yu, 2025). Invitations to participate were distributed through professional mathematics teacher associations, educational administrators, and online teacher communities. The online nature of the survey may have introduced selection bias, potentially favoring teachers with greater digital literacy and access to technology. These sampling limitations should be considered when interpreting the results, as the sample may not be fully representative of all mathematics competition teachers in China.

### 4.2. Sample Characteristics Analysis

The survey participants (N = 289) demonstrated several notable characteristics. Among the key demographics, three patterns are particularly worth highlighting: First, most participants were highly experienced educators, with 81.31% having more than 5 years of teaching experience and 84.08% having over 3 years of competition teaching experience. Second, there was a significant institutional concentration, with 63.32% of participants being from key middle schools. The detailed demographic characteristics are presented in Table 1.

### 4.3. Measurement Model Analysis

Effect sizes were reported using multiple indicators: R^2^ for variance explained and Cohen’s d for standardized mean differences. Following Cohen’s (1988) guidelines, d values of 0.2, 0.5, and 0.8 represented small, medium, and large effects, respectively, while R^2^ values of 0.01, 0.09, and 0.25 represented small, medium, and large effects, respectively.

#### 4.3.1. Descriptive Statistical Analysis

A descriptive statistical analysis revealed the overall performance levels of the measurement constructs. Table 1 presents descriptive statistics for all 28 measurement items, including sample size, mean values, standard deviations, and normality test results.

The analysis revealed several key patterns. The teaching engagement (TE) construct demonstrated notably high scores, with “I invest sufficient time in lesson preparation and research” (TE1) averaging 5.86 (SD = 1.049) and “I am enthusiastic about online teaching” (TE2) averaging 5.78 (SD = 1.048). The understanding of gifted students (UGS) construct also showed strong performance, with means ranging from 5.47 to 5.60 and standard deviations between 1.080 and 1.136.

The Shapiro–Wilk test was conducted to assess normality at α = 0.05. All variables demonstrated significant departures from normality (*p* < 0.001), with skewness ranging from −0.85 to 0.42 and kurtosis ranging from −0.56 to 1.23. The descriptive statistics and normality test results are presented in Table 2. This non-normal distribution of the measurement data justified our choice of Partial Least Squares Structural Equation Modeling (PLS-SEM) for subsequent analyses.

#### 4.3.2. Reliability and Validity Testing

Structural Equation Modeling research in social sciences also requires reliability and validity assessment of its measurements, as practiced by Lin and Yu (2025) and Wang et al. (2024b). The reliability testing results demonstrated good internal consistency in the model (Table 3). Specifically, Cronbach’s α coefficients for all seven constructs exceeded 0.85, and the composite reliability (CR) values exceeded 0.90, significantly higher than the accepted threshold of 0.70. Factor loadings ranged from 0.801 to 0.888, and AVE values (0.694–0.754) exceeded the 0.50 benchmark, confirming convergent validity.

The sustainable development construct showed the highest reliability (α = 0.892, CR = 0.925). Competition teaching professionalism (α = 0.855, CR = 0.901) and online teaching efficacy (α = 0.881, CR = 0.918) also demonstrated strong internal consistency. These results established a robust foundation for structural model analysis.

Regarding reliability assessments, DeVellis (2016) and contemporary standards for high-stakes research suggest that a scale or questionnaire should demonstrate Cronbach’s α coefficients above 0.85 to be considered highly reliable. In this study’s formal questionnaire, Cronbach’s α reliability coefficients for all factors and the overall scale exceeded 0.85, indicating excellent reliability levels that met the current rigorous standards for high-stakes research. For validity assessment, following Fornell and Larcker’s (1981) recommendations, CR values greater than 0.7 and AVE values greater than 0.5 indicated good convergent validity. In this research, all constructs’ CR values ranged between 0.901 and 0.925, and the AVE values ranged between 0.696 and 0.754, significantly exceeding the recommended standards, confirming the good convergent validity of the measurement model. Additionally, the factor loadings for CTP, OTE, SD, TA, TE, TP, and UGS all exceeded 0.5, further confirming the representativeness of the latent variable measurement items.

Three methods were employed to assess discriminant validity, as practiced by Deng and Yu (2023). First, the relationship between the square roots of the AVE values and the inter-construct correlation coefficients was examined. The results showed that the square root values of the AVE for all the constructs were greater than their correlation coefficients with the other constructs, indicating good discriminant validity between the constructs.

Table 4 presents the discriminant validity testing results. First, the square root of the AVE values on the diagonal were compared with the inter-construct correlations. All constructs showed AVE square root values greater than their correlations with the other constructs, supporting their discriminant validity.

Second, a heterotrait–monotrait ratio (HTMT) analysis revealed that all the values were below 0.50 (see Table 5), which was well within the conservative threshold of 0.90 recommended by Hair et al. (2016) for establishing discriminant validity. While the current standard accepts HTMT values up to 0.90, our observed values being substantially lower (all below 0.50) provided particularly strong evidence of discriminant validity between the constructs, exceeding typical requirements for establishing construct distinctiveness.

According to Hair et al. (2016), smaller HTMT values lead to better discrimination across variables. To meet the criterion of discriminant validity, the HTMT values should not exceed 0.900. The HTMT values between CTP, OTE, SD, TA, TE, TP, and UGS were all below 0.90, indicating that the discriminant validity met the criteria and could be further analyzed during the structural modeling process.

Finally, a cross-loading analysis confirmed that all the measurement items showed significantly higher loadings on their corresponding constructs (in bold) than on other constructs (with CTP items loading between 0.802 and 0.865 on their construct, OTE items between 0.833 and 0.877, SD items between 0.850 and 0.888, TA items between 0.825 and 0.871, TE items between 0.832 and 0.873, TP items between 0.801 and 0.855, and UGS items between 0.832 and 0.871) (Table 6).

#### 4.3.3. Collinearity Test

The collinearity among latent variables was examined through variance inflation factor (VIF) values. Table 7 presents the VIF values for all the measurement items. According to Hair et al. (2016), VIF values greater than 5.00 indicate potential collinearity issues among structural model variables. The analysis revealed that all the VIF values ranged from 1.787 to 2.569, well below the threshold of 5.00, confirming the absence of collinearity issues among the model variables.

To sum up the measurement model of the current study, measuring items had satisfactory internal consistency and reliability, and the variables were statistically distinguishable. Both the items and variables were exempted from collinearity issues. The preconditions for structural relationship analysis were all met.

#### 4.3.4. Measurement Invariance Test

To ensure measurement equivalence across different groups, this study conducted configural invariance, metric invariance, and scalar invariance tests. The results demonstrated the good cross-group equivalence of the measurement model (ΔCFI < 0.01), providing a foundation for the subsequent multi-group comparison analyses.

### 4.4. Structural Model Analysis

#### 4.4.1. Path Analysis

**Figure 2:**
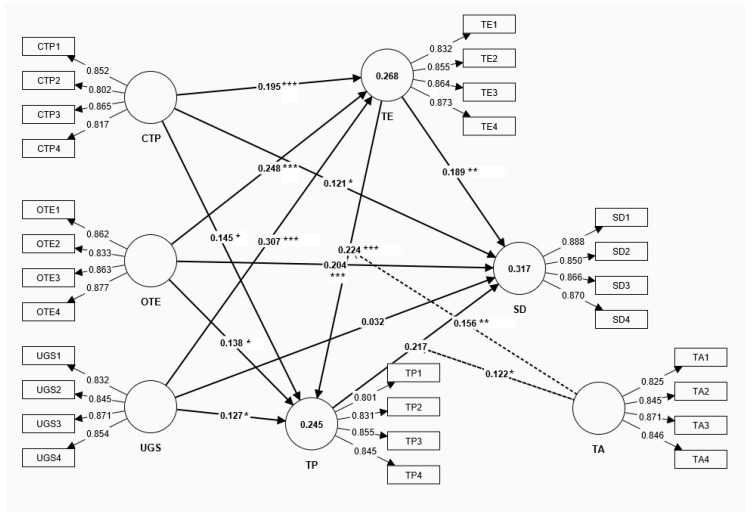
The established structural model with coefficients. *Note*. CTP: Competition teaching professionalism. OTE: Online teaching efficacy. UGS: Understanding of gifted students. TE: Teaching engagement. TP: Teaching practice. SD: Sustainable development. TA: Teacher adaptability. * *p* < 0.05, ** *p* < 0.01, and *** *p* < 0.001.

The bootstrap method (with 5000 resamples) was employed to test the significance of the path coefficients. Figure 2 illustrates the path analysis results of the overall model.

The path analysis (Table 8. Direct paths). revealed the significant direct effects of teacher capabilities on sustainable development (SD). Competition teaching professionalism (CTP) showed a significant positive influence (β = 0.121, 95% CI [0.009, 0.233], t(287) = 2.144, *p* = 0.032, R^2^ = 0.015, d = 0.25). Online teaching efficacy (OTE) demonstrated a strong positive predictive effect (β = 0.204, t = 3.754, *p* < 0.001, R^2^ = 0.042, d = 0.42). However, the understanding of gifted students (UGS) did not exhibit a significant direct effect on sustainable development (SD) (β = 0.032, t = 0.588, *p* = 0.557, R^2^ = 0.001).

Based on Cohen’s (1988) effect size criteria, the path effects were systematically analyzed. Strong effects (β > 0.35) were found in UGS’s influence on teaching engagement (β = 0.307, t = 6.644, *p* < 0.001, R^2^ = 0.094, d = 0.65). Moderate effects (0.15 < β < 0.35) were observed in several relationships: OTE’s influence on teaching engagement (β = 0.248, t = 4.965, *p* < 0.001, R^2^ = 0.061, d = 0.51), TP’s effect on sustainable development (SD) (β = 0.217, t = 4.104, *p* < 0.001), TE’s impact on TP (β = 0.224, t = 3.488, *p* < 0.001), and TE’s influence on sustainable development (SD) (β = 0.189, t = 2.871, *p* = 0.004). Weak effects (β < 0.15) were noted in CTP’s direct influence on sustainable development (SD) (β = 0.121, t = 2.144, *p* = 0.032).

#### 4.4.2. Model Fit Assessment

Multiple indicators were used to evaluate the model fit. The absolute fit indices showed satisfactory results: the SRMR (standardized root mean square residual) = 0.062, which was significantly below the critical threshold of 0.08; RMS_theta = 0.125, approaching the recommended value of 0.12; and d_ULS = 1.842 and d_G = 0.763, both within acceptable ranges.

The incremental fit indices demonstrated a good model fit: the NFI (Normed Fit Index) = 0.921, exceeding the recommended threshold of 0.90, and the RFI (Relative Fit Index) = 0.908, indicating a good relative fit. The parsimony fit indices (PGFI = 0.684; PNFI = 0.677) suggested that the model maintained good explanatory power while remaining parsimonious.

#### 4.4.3. Mediation Effects Analysis

A bootstrap analysis (n = 5000) was conducted to examine both simple and chain mediation effects(Table 9). The results confirmed the significant mediating roles of teaching engagement and teaching practice on the relationship between teacher capabilities and sustainable development (SD). Specifically, the hypotheses in Table 9 were supported at *p* < 0.05.

The bootstrap analysis (n = 5000) revealed that, for simple mediation effects, the indirect effect of CTP→TE→SD was 0.037 (95% CI [0.002, 0.072], SE = 0.018, t = 2.080, *p* = 0.038, κ^2^ = 0.045). Teaching engagement significantly mediated the relationship between competition teaching professionalism (CTP) and sustainable development (SD) (β = 0.037, t = 2.080, *p* < 0.05). Similarly, teaching practice showed significant mediation effects between competition teaching professionalism (CTP) and sustainable development (SD) (β = 0.031, t = 2.009, *p* < 0.05).

#### 4.4.4. Moderation Effect Analysis

The product indicator approach was employed to test teacher adaptability’s moderating effects (Table 10). The results confirmed the significant positive moderating effects of teacher adaptability on the relationships between teaching engagement and teaching practice (β = 0.156, t = 2.661, *p* < 0.01) and between teaching practice and sustainable development (SD) (β = 0.122, t = 2.252, *p* < 0.05).

These results indicated that higher teacher adaptability strengthened both the positive effect of teaching engagement on teaching practice and the positive influence of teaching practice on students’ sustainable development (SD). This finding highlighted the crucial role of teachers’ adaptive capabilities in online mathematical competition education.

#### 4.4.5. Robustness Test

The PLS-SEM method (Dijkstra & Henseler, 2015) was employed to conduct robustness tests. The results confirmed the stability of the model estimates through the following indicators: SRMR = 0.062 (below the critical value of 0.08), NFI = 0.921 (exceeding 0.90), and GOF = 0.526 (exceeding 0.36). These findings further validated the reliability of the research results.

### 4.5. Model Predictive Power Assessment

A comprehensive evaluation of the model’s predictive capability was conducted using multiple indicators (Table 11). An effect size (f^2^) analysis revealed varying degrees of influence among the predictor variables. The understanding of gifted students demonstrated the strongest effect on teaching engagement (f^2^ = 0.119). Online teaching efficacy showed good predictive power for teaching engagement (f^2^ = 0.078), and competition teaching professionalism (CTP) also exhibited a meaningful prediction of teaching engagement (f^2^ = 0.049).

A coefficient of determination (R^2^) analysis demonstrated moderate explanatory power for all three endogenous variables (Table 12). Specifically, the model explained 31.7% of the variance in sustainable development (R^2^ = 0.317), 26.8% in teaching engagement (R^2^ = 0.268), and 24.5% in teaching practice (R^2^ = 0.245), indicating a satisfactory explanatory capability.

Predictive relevance was assessed through Stone–Geisser’s Q^2^ value (Table 13). The results showed positive Q^2^ values for sustainable development (Q^2^ = 0.225), teaching engagement (Q^2^ = 0.190), and teaching practice (Q^2^ = 0.158), all at moderate levels, confirming the model’s predictive relevance.

Collectively, these indicators substantiated the model’s reliable predictive capability and practical guidance value. The model effectively explained how teachers’ capabilities influenced students’ sustainable development through teaching processes, revealing the moderating role of teacher adaptability. These findings could provide valuable insights for enhancing the effectiveness of online mathematical competition education.

## 5. Discussion

### 5.1. Key Research Findings

#### 5.1.1. Differential Impact of Teacher Competencies

This study reveals that teacher competencies have varying impacts on the teacher-rated sustainable development of students in online mathematics competition education. Specifically, online teaching efficacy (OTE) has a strong direct effect (H1a, β = 0.204, *p* < 0.001), while competition teaching professionalism (CTP) has a weaker but still significant direct effect (H1b, β = 0.121, *p* = 0.032). This finding contrasts with traditional classroom teaching research, where teachers’ professional knowledge typically has a more important role (Ball et al., 2008). Presumably, this results from increased psychological distance and reduced anxiety caused by teacher authority. Pedagogical and content knowledge also facilitated teaching reflection on video-recorded face-to-face teaching practice (Kulgemeyer & Riese, 2018). However, in online environments, technological mediation introduces new variables that influence how professional knowledge is conveyed and applied, highlighting the crucial role of teachers’ online teaching capabilities in maintaining educational effectiveness. This explanation aligns with the recent findings of S. Huang et al. (2022), who emphasized the importance of online teaching efficacy and self-efficacy in engagement. Furthermore, Dong et al. (2023) found that teacher adaptability significantly moderated changes in online teaching self-efficacy, particularly during technological transitions.

While CTP shows a relatively weak direct effect on students’ sustainable development (H1a, β = 0.121), its indirect effects through mediating pathways (i.e., H5a and H5b; total indirect effect = 0.068) remain significant and moderate. This indicates that teachers’ professional knowledge influences students through not only direct teaching interactions but also dynamic interactions in the educational process and students’ learning methods. The transformation of teacher competencies requires a process of teacher engagement and practice, highlighting how technology intervention affects the transmission and application of teacher knowledge. Contemporary perspectives on the psychological dynamics of learning and interaction in mathematics learning can support such an explanation (e.g., Abrahamson & Sánchez-García, 2016).

#### 5.1.2. Role of Teachers’ Understanding of Gifted Students

Our analysis reveals an unexpected finding regarding the relationship between teachers’ understanding of gifted students (UGS) and the teacher-rated sustainable development of learners. Contrary to our initial hypothesis (H1c), which proposed a direct positive effect, the UGS showed an insignificant direct effect on students’ sustainable development (β = 0.032, *p* = 0.557). However, this finding provides important theoretical insights about the nature of teacher knowledge translation in online educational environments.

This apparent contradiction between our hypothesis and findings can be explained through several theoretical mechanisms. First, the lack of a direct effect suggests that teacher knowledge requires active operationalization through specific pedagogical approaches to be effective in online environments. This aligns with Mishra and Koehler’s (2006) framework, which emphasizes that effective teaching requires the integration of knowledge through practical implementation rather than the mere possession of understanding.

While the direct effect was not significant, our analysis revealed that the UGS demonstrates substantial influence through indirect pathways. Specifically, the UGS shows a strong positive influence on teaching engagement (H2c, β = 0.307, *p* < 0.001), which in turn affects teaching practice (H2d, β = 0.127, *p* = 0.020). These sequential relationships culminate in significant total indirect effects on sustainable development (H4c, β = 0.015, *p* = 0.018). This pattern suggests that the impact of teachers’ understanding manifests primarily through its implementation in teaching processes rather than through direct transmission.

The transformation of theoretical knowledge into practical outcomes appears to require specific mediating mechanisms in online environments. This finding extends Subotnik et al.’s (2011) comprehensive development model by highlighting how teachers’ understanding of gifted students must be actively transformed into specific teaching strategies and practices to be effective. Furthermore, this result suggests that future teacher development programs should focus not only on building teachers’ understanding of gifted students but also on developing their capacity to operationalize this knowledge through effective online teaching practices.

#### 5.1.3. Role of Mediating Mechanisms

Teaching engagement and teaching practice play crucial mediating roles in transforming teacher competencies into educational outcomes. Teachers’ understanding of gifted students influences student development through the sequential mediation of teaching engagement (H2c, β = 0.307, *p* < 0.001) and teaching practice (H3c, β = 0.127, *p* = 0.020). This chain-mediated effect emphasizes the central position of the teaching process in knowledge transformation. This research has established that the understanding of gifted students (UGS) indirectly affects student development through the chain-mediated pathways of teaching engagement and practice (H4c, β = 0.015, *p* = 0.018), indicating that traditional gifted education theories need to be transformed through interactive design and dynamic feedback mechanisms in online teaching.

#### 5.1.4. Moderating Effect of Teacher Adaptability

Our research revealed a novel dual moderating mechanism of teacher adaptability in online mathematics competition education. This mechanism manifests through two distinct pathways of enhancement: process enhancement and outcome enhancement.

The process enhancement pathway (H6a, β = 0.156, *p* = 0.008) strengthens the connection between teaching engagement and teaching practice. This finding suggests that adaptable teachers more effectively translate their engagement into coherent teaching practices through the flexible implementation and responsive modification of instructional strategies. This addresses what Mishra and Koehler (2006) identified as the “wicked problem” of technology integration—the complex challenge of dynamically balancing technological, pedagogical, and content knowledge in constantly evolving environments.

The outcome enhancement pathway (H6b, β = 0.122, *p* = 0.024) amplifies the positive impact of teaching practice on sustainable development. This pathway is particularly significant in mathematics competition education, where problems often have multiple solution approaches requiring teachers to adapt their guidance based on students’ thinking processes. This finding aligns with Leikin’s (2011) research on multiple-solution tasks, suggesting that teacher adaptability plays a crucial role in supporting the development of students’ mathematical creativity through responsive feedback mechanisms.

The interaction between these two enhancement pathways creates a synergistic effect that promotes overall teaching quality. This dual mechanism extends significantly beyond König et al.’s (2020) single-pathway model by revealing how teacher adaptability creates a comprehensive enhancement effect throughout the teaching process. It helps explain why some teachers maintain effectiveness during technological transitions while others struggle despite similar professional knowledge and technological skills. This suggests that professional development programs should focus on enhancing adaptability across multiple dimensions rather than merely building technological or pedagogical knowledge in isolation.

#### 5.1.5. Demographic Analysis

The analysis of demographic factors revealed several significant patterns. Our participants demonstrated distinct characteristics that warrant careful consideration. Most participants were highly experienced educators. While this depth of experience provides valuable insights into established teaching practices, it may limit our understanding of the challenges faced by novice teachers in online mathematics competition education. Although experiences may allow teachers to more deeply dedicate themselves to the teaching process, mechanisms have also illustrated that high relatedness to students could result in a rapid decline in teachers’ psychological well-being (Collie & Martin, 2023). Therefore, it is reasonable to assume that the contribution of teaching experience to teaching effectiveness may face a plateau effect.

The institutional distribution in our sample showed significant concentration, with 63.32% of participants being from key middle schools. While these schools typically have superior resources and more experienced teaching staff, this concentration may introduce systematic biases into our findings. Key middle schools typically have better technological infrastructure and support systems, resulting in participants potentially overestimating the feasibility of online teaching implementation in resource-constrained environments.

Furthermore, the observed teaching engagement levels (mean = 5.86) require careful interpretation, as they may reflect the advantaged position of key middle schools rather than the broader reality of mathematics competition education. Regular middle schools, comprising 7.61% of our sample, may require additional technological infrastructure and professional development support to maintain competitive standards in online mathematics competition education.

Although demographic features are not examined explicitly in the current study, their impacts have been noticed in mathematics education for decades (e.g., Borko & Livingston, 1989; R. Huang & Li, 2012; Wardat et al., 2024). Based on the current sample, the current study contributes to the research area with a particular set of demographic features in mathematics gifted education, a less explored direction. Continued efforts are needed to elucidate the complex cognitive, affective, and behavioral mechanisms of teachers and students in such contexts.

### 5.2. Theoretical Contributions

This research advances existing educational theory in three significant aspects:

First, we establish an innovative theoretical framework integrating teacher competencies and student development. This framework demonstrates how different dimensions of teacher competencies—competition teaching professionalism, online teaching efficacy, and the understanding of gifted students—influence students’ sustainable development through teaching processes. Notably, our findings reveal that online teaching efficacy shows a stronger direct impact (β = 0.204) on students’ sustainable development than competition teaching professionalism (CTP) (β = 0.121). This challenges traditional assumptions about knowledge transmission methods (Ball et al., 2008), highlighting the crucial role of technology-mediated instruction in educational effectiveness.

Second, we uncover a novel chain-mediation mechanism in gifted education. While teachers’ understanding of gifted students shows insignificant direct effects (β = 0.032), it is effectively transformed into educational outcomes through the mediating pathways of teaching engagement and teaching practice (β = 0.015). This extends Subotnik et al.’s (2011) gifted education theory by highlighting the specific transformation mechanisms through which teachers’ understanding of gifted students’ needs manifests in educational outcomes.

Third, we propose an innovative “Dual Enhancement Model” of teacher adaptability in online mathematics competition education. The model demonstrates how adaptability’s influence dynamically varies across different teaching phases, and it explains the varying levels of effectiveness among teachers during technological transitions.

### 5.3. Practical Implications

Our empirical findings provide insights that may inform approaches to enhancing online mathematics competition education. Based on our research results, we propose several potential recommendations that should be considered in light of the study’s limitations:

First, the results suggest that educational institutions might benefit from establishing professional development programs that address multiple dimensions of teacher competency. Given that both competition teaching professionalism and online teaching efficacy demonstrated positive relationships with sustainable development, institutions should consider developing comprehensive training programs that strengthen teachers’ mathematical competition knowledge while simultaneously enhancing their digital teaching capabilities. These programs could include collaborative problem-solving workshops, mentoring from experienced competition teachers, and hands-on training with online educational platforms and tools. However, these recommendations should be implemented with a careful consideration of institutional contexts and resources, as our study primarily reflects the experiences of teachers from well-resourced key middle schools in China. Teachers’ well-being should be taken into consideration in their close association with students. For example, researchers have demonstrated that teachers’ sense of relatedness with students could accelerate the decline in teachers’ psychological well-being over time (Collie & Martin, 2023).

Second, educational institutions should implement differentiated support strategies based on school type and existing resources. In the digital reform of education, teachers have actively adopted emerging technologies, like AI, and researchers have also identified reasons behind teachers’ unwillingness to adopt AI to assist student learning, which appeals to further teacher training and student support from institutions (Collie & Martin, 2025). For key middle schools, the focus should be on advancing sophisticated pedagogical approaches and fostering higher-order thinking skills. These institutions should establish professional learning communities that facilitate systematic knowledge-sharing and collaborative innovation among educators. This approach enables experienced teachers to mentor colleagues while promoting the continuous evolution of teaching practices. For regular middle schools, the emphasis should be on strengthening fundamental infrastructure and building core competencies. These institutions would benefit from comprehensive support systems that include technical resources, structured training programs, and standardized teaching protocols. The focus should be on developing adaptable teaching methodologies that can accommodate diverse student needs while maintaining high educational standards. Training institutions should focus on developing standardized online teaching procedures while maintaining the flexibility to adapt to different student needs and learning paces. It is also worth noting that our research topic should be extended to investigate whether contextual factors like school types would drastically change our conclusions.

Third, institutions must establish robust evaluation and support systems to enhance teaching effectiveness. This should begin with the development of comprehensive teacher evaluation mechanisms that assess both competition teaching expertise and online teaching capabilities. The evaluation criteria should include measures of student engagement, learning outcomes, and teaching innovation (see Wafudu & Bin Kamin, 2024, for an example of teaching quality assurance in vocational education). Furthermore, institutions should create resource-sharing platforms that facilitate the exchange of teaching materials, methodologies, and best practices among teachers. These platforms should include both synchronous and asynchronous communication channels to support ongoing professional dialogue and collaboration. Additionally, institutions should implement incentive mechanisms that recognize and reward teaching innovation and effectiveness, particularly in the online environment.

Fourth, given the significant moderating role of teacher adaptability (β = 0.156, *p* = 0.008), institutions should prioritize developing teachers’ adaptive capabilities through structured support systems. This can be achieved by establishing regular professional learning communities where teachers can share experiences, challenges, and solutions related to online mathematics competition teaching. These communities should meet regularly to discuss emerging challenges and innovative solutions in online teaching. Additionally, institutions should provide structured feedback mechanisms that help teachers identify areas for adaptation and improvement in their teaching practices. This feedback should come from multiple sources, including peer observations, student evaluations, and self-reflection protocols.

### 5.4. Limitations and Future Research Directions

While this study makes significant contributions to understanding online mathematics competition education, several limitations should be acknowledged, and corresponding future research directions can be identified.

A significant limitation of this study is the absence of comparison groups, which restricts the interpretability of our findings. The current study only focused on teachers from gifted education, and the sampling in the current study relied on convenient samples accessible to the researchers, which indicates that their eligibility for the current study relied mostly on the participants’ self-reporting. This limitation prevents us from making definitive claims about whether the relationships identified are unique to online mathematics competition education or might apply equally to other educational contexts. Future research should address this limitation through comparative designs that include multiple teacher groups and teaching formats, allowing for a more nuanced understanding of context-specific effects.

For research practice, researchers should expand the population under investigation to include regular and offline mathematics learners at comparable educational stages, where potential moderators like student types (gifted vs. non-gifted) and learning media (online vs. offline) could be explored. In online learning, timing is usually explored to reflect whether synchronous and asynchronous teaching, feedback, and collaboration demonstrate substantial differences (e.g., Zeng & Luo, 2024). For example, asynchronous learning was found to be more effective than a synchronous mode (Zeng & Luo, 2024), which should be considered in future research to examine our research contexts and beyond. Timing may allow students to better understand their learning content and solve problems when it comes to abstract and complex problems in mathematics competition learning. Such contextual factors were found to be critical predictors of academic achievements, for example, socioeconomic factors (Collie & Martin, 2024; Collie & Hascher, 2024). Introducing such factors into investigations and explanations should help future researchers make contributions to understanding contextualized learner and teacher populations.

However, such designs, especially those requiring Structural Equation Modeling techniques that help model multiple variables, should entail a more balanced sample consisting of the abovementioned sub-groups. It will also be interesting to explore the relevant research themes through more traditional quasi-experimental designs to test if interventions can improve teachers’ and students’ competencies. This limitation should not undermine our innovative contributions made by exploring a specific context in gifted education.

Another important limitation concerns our measurement of the outcome variable “sustainable development”. This variable was measured through teacher self-reports, which are subject to the discrepancy between learning gains and observers’ ratings, rather than through the independent assessment of student outcomes. Thus, this approach introduces potential bias, as teachers may overestimate their students’ development or may be influenced by their perceptions of their own teaching effectiveness. Furthermore, each dimension of student development (performance, problem-solving, creative thinking, and motivation) was measured with only a single item, limiting the depth and reliability of these assessments.

Researchers have noted the discrepancy between learners’ ratings and their actual gains (e.g., Lin et al., 2023), but most admit the value of establishing perceptual data, encouraging the incorporation of more objective measurements. This issue is similar to our situation, whether it be self- or others’ observation, since measuring the predictors and outcomes from the same perspective may involve common method bias. However, given the advantages of questionnaire measurements, researchers must be aware of the potential risk and interpret our findings with caution to strike a balance among methodologies. Future research should incorporate more robust outcome measures, including direct assessments of student performance, multi-item scales for each dimension of development, and the longitudinal tracking of student outcomes. Ideally, such research would also include student self-reports and independent evaluations to provide a more comprehensive and objective assessment of sustainable development. Qualitative results are also welcomed to consolidate the perceptual outcomes of digital gifted education explored in this study.

The cross-sectional nature of our study presents another limitation, as it may prevent us from establishing causal relationships between the variables examined. The data represent a single point in time, making it impossible to determine whether teacher competencies lead to effective teaching processes and student development, or whether successful experiences with students might enhance teachers’ sense of competence and engagement. Additionally, our reliance on self-report measures introduces potential social desirability bias, as teachers may report their competencies, practices, and students’ development in an overly positive light. These methodological limitations suggest that our findings should be interpreted as correlational rather than causal, and that the practical implications derived from these findings should be viewed as preliminary until confirmed by more robust longitudinal and mixed-methods research.

The sample representation is also limited. Our study primarily drew participants from key middle schools in China, with 63.3% of the participants coming from key middle schools. This gender and institutional concentration may limit the generalizability of our findings to other contexts. However, as reflected in Lin et al. (2023), the psychological mechanisms revealed in our particular research context may bring enlightenment and be extended to broader contexts. Future research should increase representation from regular middle schools and training institutions to ensure broader institutional diversity in the sample. Additionally, researchers should consider conducting comparative studies across different regions and school types to understand how contextual factors influence teaching effectiveness.

Another significant limitation is that we measured overall teaching experience rather than specifically assessing teachers’ experience with online teaching. The transition from face-to-face to online teaching involves substantial adaptation, and teachers’ level of experience with online instruction likely influences their effectiveness in this environment. Future research should explicitly measure both overall teaching experience and online teaching experience as separate variables, examining how the relationship between teacher competencies and student outcomes might be moderated by familiarity with online teaching formats. This would provide more nuanced insights into the development of teaching effectiveness in digital environments.

Moreover, our study focused exclusively on mathematics competition education for gifted students in online environments. Future research should explore how these same teaching processes operate in regular mathematics education and face-to-face contexts. Such comparative studies would help identify which aspects of our findings are specific to online mathematics competition education and which may be generalizable to broader educational contexts. This expansion would contribute to a more comprehensive understanding of effective teaching processes across various educational settings and student populations.

Despite these limitations, our study provides valuable insights into the dynamics of online mathematics competition education and lays the groundwork for future research in this important field. Subsequent studies may consider not only addressing these limitations but also exploring some emerging areas, such as the role of artificial intelligence in mathematics competition education, the impact of various online teaching platforms on learning outcomes, and the development of innovative assessment methods for online mathematics competition education (e.g., Almarashdi et al., 2024). Reflecting on another notable branch of research into educational technology, students’ and teachers’ intentions to use technology, their technostress, attitudes, and other influencing factors should receive future attention (e.g., Dai et al., 2024; Wang et al., 2025; Lin & Yu, 2024), especially when emerging technologies are prepared for pedagogical designs. Such research should benefit technological integration into mathematics gifted education.

## Figures and Tables

**Table 1:** Demographic information of participants.

Survey Item	Option	Count	Percentage
Gender	Male	261	90.31%
	Female	28	9.69%
Age	Under 25	23	7.96%
	26–35	109	37.72%
	36–45	106	36.68%
	Over 46	51	17.65%
Teaching Experience	Under 5 years	54	18.69%
	6–10 years	86	29.76%
	11–15 years	40	13.84%
	Over 15 years	109	37.72%
Competition Teaching Experience	Under 3 years	46	15.92%
	3–5 years	41	14.19%
	6–10 years	88	30.45%
	Over 10 years	114	39.45%
School Type	Key middle school	183	63.32%
	Regular middle school	22	7.61%
	Training institution	84	29.07%

Note. School types in the Chinese education system are differentiated as follows: Key middle schools are selective public schools that receive additional government funding and typically have stronger academic programs, particularly in mathematics and sciences. They are roughly comparable to magnet schools in the USA or grammar schools in the UK. Regular middle schools are standard public schools that follow the national curriculum. Training institutions are private educational organizations that offer specialized after-school programs, similar to learning centers or supplementary education providers in Western countries.

**Table 2:** Descriptive statistics of measurement items.

Variable	Average Value	Standard Deviation	Shapiro–Wilk	
			Statistics	Significance
CTP1	5.42	1.077	0.906	<0.001
CTP2	5.06	1.091	0.921	<0.001
CTP3	5.43	1.085	0.899	<0.001
CTP4	4.72	1.162	0.929	<0.001
OTE1	4.91	1.172	0.930	<0.001
OTE2	4.91	1.200	0.931	<0.001
OTE3	5.13	1.195	0.923	<0.001
OTE4	4.68	1.203	0.933	<0.001
UGS1	5.55	1.120	0.897	<0.001
UGS2	5.60	1.136	0.892	<0.001
UGS3	5.52	1.080	0.902	<0.001
UGS4	5.47	1.130	0.904	<0.001
TE1	5.86	1.049	0.862	<0.001
TE2	5.78	1.048	0.873	<0.001
TE3	5.56	1.117	0.896	<0.001
TE4	5.55	1.117	0.894	<0.001
TP1	5.51	1.080	0.902	<0.001
TP2	5.11	1.085	0.917	<0.001
TP3	5.49	1.048	0.895	<0.001
TP4	5.58	1.054	0.890	<0.001
SD1	5.40	1.120	0.906	<0.001
SD2	5.44	1.157	0.906	<0.001
SD3	5.46	1.175	0.903	<0.001
SD4	5.44	1.177	0.906	<0.001
TA1	5.67	1.060	0.888	<0.001
TA2	5.34	1.144	0.911	<0.001
TA3	5.57	1.084	0.896	<0.001
TA4	5.63	1.069	0.891	<0.001

Note. CTP: Competition teaching professionalism. OTE: Online teaching efficacy. UGS: Understanding of gifted students. TE: Teaching engagement. TP: Teaching practice. SD: Sustainable development. TA: Teacher adaptability.

**Table 3:** Reliability and convergent validity test results.

Dimension	Item	Factor Loading	α	rho_a	CR	AVE
CTP	CTP1	0.852	0.855	0.867	0.901	0.696
	CTP2	0.802				
	CTP3	0.865				
	CTP4	0.817				
OTE	OTE1	0.862	0.881	0.887	0.918	0.737
	OTE2	0.833				
	OTE3	0.863				
	OTE4	0.877				
SD	SD1	0.888	0.892	0.898	0.925	0.754
	SD2	0.850				
	SD3	0.866				
	SD4	0.870				
TA	TA1	0.825	0.871	0.890	0.910	0.718
	TA2	0.845				
	TA3	0.871				
	TA4	0.846				
TE	TE1	0.832	0.878	0.879	0.916	0.733
	TE2	0.855				
	TE3	0.864				
	TE4	0.873				
TP	TP1	0.801	0.853	0.855	0.901	0.694
	TP2	0.831				
	TP3	0.855				
	TP4	0.845				
UGS	UGS1	0.832	0.873	0.876	0.913	0.724
	UGS2	0.845				
	UGS3	0.871				
	UGS4	0.854				

Note. CTP = competition teaching professionalism; OTE = online teaching efficacy; SD = sustainable development; TA = teacher adaptability; TE = teaching engagement; TP = teaching practice; UGS = understanding of gifted students; α = Cronbach’s alpha; CR = composite reliability; AVE = Average Variance Extracted.

**Table 4:** Discriminant validity test results using the Fornell–Larcker criterion.

	CTP	OTE	SD	TA	TE	TP	UGS
CTP	0.834						
OTE	0.182	0.859					
SD	0.286	0.390	0.869				
TA	0.026	0.088	0.133	0.847			
TE	0.294	0.356	0.417	0.073	0.856		
TP	0.265	0.304	0.397	0.144	0.374	0.833	
UGS	0.174	0.235	0.252	0.021	0.399	0.294	0.851

Note. CTP: Competition teaching professionalism. OTE: Online teaching efficacy. UGS: Understanding of gifted students. TE: Teaching engagement. TP: Teaching practice. SD: Sustainable development. TA: Teacher adaptability.

**Table 5:** Discriminant validity test results using HTMT analysis.

	CTP	OTE	SD	TA	TE	TP	UGS
CTP							
OTE	0.210						
SD	0.321	0.435					
TA	0.058	0.101	0.144				
TE	0.330	0.401	0.465	0.093			
TP	0.310	0.345	0.449	0.163	0.431		
UGS	0.198	0.268	0.279	0.072	0.455	0.340	

Note. CTP: Competition teaching professionalism. OTE: Online teaching efficacy. UGS: Understanding of gifted students. TE: Teaching engagement. TP: Teaching practice. SD: Sustainable development. TA: Teacher adaptability.

**Table 6:** Discriminant validity test results using cross-loading analysis.

	CTP	OTE	SD	TA	TE	TP	UGS
CTP1	0.852	0.153	0.271	0.010	0.299	0.215	0.151
CTP2	0.802	0.195	0.216	−0.018	0.170	0.191	0.093
CTP3	0.865	0.140	0.240	0.083	0.291	0.237	0.164
CTP4	0.817	0.130	0.220	−0.002	0.196	0.240	0.163
OTE1	0.209	0.862	0.368	0.095	0.332	0.306	0.194
OTE2	0.146	0.833	0.332	0.044	0.321	0.230	0.244
OTE3	0.136	0.863	0.283	0.097	0.263	0.232	0.168
OTE4	0.126	0.877	0.344	0.066	0.297	0.266	0.199
SD1	0.310	0.351	0.888	0.085	0.443	0.371	0.295
SD2	0.285	0.321	0.850	0.114	0.323	0.355	0.176
SD3	0.182	0.339	0.866	0.175	0.366	0.349	0.197
SD4	0.204	0.343	0.870	0.090	0.301	0.295	0.193
TA1	0.037	0.084	0.032	0.825	0.038	0.115	0.030
TA2	0.045	0.035	0.152	0.845	−0.005	0.095	−0.068
TA3	0.029	0.092	0.111	0.871	0.079	0.160	0.047
TA4	−0.020	0.089	0.127	0.846	0.127	0.112	0.064
TE1	0.260	0.332	0.301	0.046	0.832	0.296	0.378
TE2	0.260	0.325	0.392	0.053	0.855	0.322	0.345
TE3	0.214	0.275	0.385	0.108	0.864	0.323	0.321
TE4	0.272	0.284	0.348	0.042	0.873	0.340	0.323
TP1	0.253	0.264	0.290	0.147	0.274	0.801	0.298
TP2	0.190	0.281	0.393	0.155	0.329	0.831	0.217
TP3	0.232	0.241	0.320	0.082	0.350	0.855	0.228
TP4	0.211	0.221	0.310	0.091	0.290	0.845	0.238
UGS1	0.107	0.184	0.174	−0.032	0.363	0.221	0.832
UGS2	0.168	0.224	0.205	0.022	0.302	0.233	0.845
UGS3	0.145	0.201	0.248	0.076	0.345	0.273	0.871
UGS4	0.172	0.193	0.224	0.000	0.346	0.268	0.854

Note. CTP: Competition teaching professionalism. OTE: Online teaching efficacy. UGS: Understanding of gifted students. TE: Teaching engagement. TP: Teaching practice. SD: Sustainable development. TA: Teacher adaptability.

**Table 7:** Collinearity test results using VIF values for measurement items.

Variable	Item	VIF	Variable	Item	VIF
CTP	CTP1	2.018	TE	TE1	2.043
	CTP2	1.931		TE2	2.204
	CTP3	2.189		TE3	2.318
	CTP4	1.921		TE4	2.445
OTE	OTE1	2.244	TP	TP1	1.787
	OTE2	2.061		TP2	1.910
	OTE3	2.474		TP3	2.159
	OTE4	2.479		TP4	2.133
SD	SD1	2.554	UGS	UGS1	1.987
	SD2	2.178		UGS2	2.188
	SD3	2.346		UGS3	2.295
	SD4	2.569		UGS4	2.128
TA	TA1	2.244			
	TA2	2.001			
	TA3	2.152			
	TA4	2.044			

Note. CTP: Competition teaching professionalism. OTE: Online teaching efficacy. UGS: Understanding of gifted students. TE: Teaching engagement. TP: Teaching practice. SD: Sustainable development. TA: Teacher adaptability. VIF: Variance inflation factor.

**Table 8:** Direct paths.

Hypothesis		*β*	Mean	STDEV	T	*p*	Hypothesis Testing Results
H1a	CTP → SD	0.121	0.123	0.057	2.144	0.032 *	Supported
H1b	OTE → SD	0.204	0.205	0.054	3.754	<0.001 ***	Supported
H1c	UGS → SD	0.032	0.034	0.055	0.588	0.557	Not Supported
H2a	CTP → TE	0.195	0.198	0.050	3.917	<0.001 ***	Supported
H2b	OTE → TE	0.248	0.248	0.050	4.965	<0.001 ***	Supported
H2c	UGS → TE	0.307	0.309	0.046	6.644	<0.001 ***	Supported
H2d	TE → TP	0.224	0.222	0.064	3.488	<0.001 ***	Supported
H2e	TE → SD	0.189	0.188	0.066	2.871	0.004 **	Supported
H3a	CTP → TP	0.145	0.146	0.061	2.361	0.018 *	Supported
H3b	OTE → TP	0.138	0.141	0.060	2.317	0.021 *	Supported
H3c	UGS → TP	0.127	0.129	0.054	2.324	0.020 *	Supported

Note. CTP: Competition teaching professionalism. OTE: Online teaching efficacy. UGS: Understanding of gifted students. TE: Teaching engagement. TP: Teaching practice. SD: Sustainable development. TA: Teacher adaptability. STDEV: Standard Deviation. * *p* < 0.05, ** *p* < 0.01, and *** *p* < 0.001.

**Table 9:** Mediation effects.

Hypothesis		*β*	Mean	STDEV	T Statistics	*p* Values	Hypothesis Testing Results
**H4a**	CTP → TE → TP → SD	0.01	0.009	0.004	2.182	0.029 *	Supported
**H4b**	OTE → TE → TP → SD	0.012	0.012	0.005	2.256	0.024 *	Supported
**H4c**	UGS → TE → TP → SD	0.015	0.015	0.006	2.374	0.018 *	Supported
**H5a**	CTP → TE → SD	0.037	0.038	0.018	2.080	0.038 *	Supported
**H5b**	CTP → TP → SD	0.031	0.032	0.016	2.009	0.045 *	Supported
**H5c**	OTE → TE → SD	0.047	0.047	0.020	2.386	0.017 *	Supported

Note. CTP: Competition teaching professionalism. OTE: Online teaching efficacy. UGS: Understanding of gifted students. TE: Teaching engagement. TP: Teaching practice. SD: Sustainable development. TA: Teacher adaptability. STDEV: Standard Deviation. * *p* < 0.05.

**Table 10:** Moderation effects.

	Hypothesis	β	Mean	STDEV	T Statistics	*p* Values	Hypothesis Testing Results
**H6a**	TA × TE → TP	0.156	0.154	0.058	2.661	0.008 **	Supported
**H6b**	TA × TP → SD	0.122	0.117	0.054	2.252	0.024 *	Supported

Note. TE: Teaching engagement. TP: Teaching practice. SD: Sustainable development. TA: Teacher adaptability. STDEV: Standard Deviation. * *p* < 0.05, ** *p* < 0.01.

**Table 11:** Effect size (f^2^) test results.

	TE	TP	SD
CTP	0.049	0.025	0.019
OTE	0.078	0.021	0.050
TE		0.048	0.036
TP			0.053
UGS	0.119	0.017	0.001
TA × TE		0.029	
TA × TP			0.018

Note. CTP: Competition teaching professionalism. OTE: Online teaching efficacy. UGS: Understanding of gifted students. TE: Teaching engagement. TP: Teaching practice. SD: Sustainable development. TA: Teacher adaptability.

**Table 12:** Coefficient of determination (R^2^) analysis results.

	R-Square	Degree
SD	0.317	Moderate
TE	0.268	Moderate
TP	0.245	Moderate

Note. TE: Teaching engagement. TP: Teaching practice. SD: Sustainable development.

**Table 13:** Predictive relevance (Q^2^) analysis results.

	SSO	SSE	Q^2^ (=1 − SSE/SSO)	Degree
CTP	1160	1160		
OTE	1160	1160		
SD	1160	898.501	0.225	Moderate
TA	1160	1160		
TE	1160	939.493	0.190	Moderate
TP	1160	976.427	0.158	Moderate
UGS	1160	1160		

Note. CTP: Competition teaching professionalism. OTE: Online teaching efficacy. UGS: Understanding of gifted students. TE: Teaching engagement. TP: Teaching practice. SD: Sustainable development. TA: Teacher adaptability.

## Data Availability

The data supporting this study’s findings are available from the authors upon reasonable request.

## Appendix A. Questionnaire on Online Education for Mathematics Competition Teachers (English Version)

Dear teacher,

This questionnaire aims to understand the online education situation of mathematics competition teachers. The questionnaire items are adapted based on the following scale:

- Teacher Professional Questions Adapted from Ball et al.’s (2008) Mathematics Teaching Knowledge (MKT) Scale
- The teaching effectiveness items are adapted from the TSES scale by Tschannen-Moran and Hoy (2001)
- Teacher participation questions adapted from Schaufeli et al.’s (2002) UWES scale

This study adopts a mixed research method combining questionnaire surveys and in-depth interviews. After completing the questionnaire, we will select some teachers for a 30–45 min in-depth interview to gain a deeper understanding of your experience and insights in online teaching. If you are willing to participate in the follow-up interview, please leave your contact information at the end of the questionnaire.

Basic Information

1. Gender: □ Male □ Female
2. Age: □ Under 25 years old □ 26–35 years old □ 36–45 years old □ Over 46 years old
3. Teaching years: □ Less than 5 years □ 6–10 years □ 11–15 years □ Over 15 years
4. Competition teaching experience: □ Less than 3 years □ 3–5 years □ 6–10 years □ Over 10 years
5. School type: □ Key high school □ Ordinary high school □ Training institution

**Main issues (using a 7-point Likert scale: 1 = completely disagree, 7 = completely agree)**

**Competition Teaching Professionalism (ξ 1)**

1. I have mastered the systematic knowledge system of mathematical competitions
2. I can design effective competition problem-solving strategies
3. I can cultivate students’ mathematical competition thinking
4. I can accurately grasp the rules and directions of competition proposition

**Online teaching effectiveness (ξ 2)**

5. I am proficient in using various online teaching platforms
6. I am able to design course content suitable for online teaching
7. I am able to effectively integrate online teaching resources
8. I am able to handle technical issues in online teaching effectively

**Understanding of Gifted Students (ξ 3)**

9. I have a deep understanding of the learning characteristics of gifted students
10. I can accurately grasp the learning needs of gifted students
11. I can provide targeted learning guidance
12. I understand the psychological characteristics of gifted students in competitive learning

**Teaching participation (η 1)**

13. I invest a lot of time in teaching preparation
14. I often reflect on and improve my teaching methods
15. I actively participate in teaching seminars and exchanges
16. I continue to monitor the latest developments in teaching

**Teaching Practice (η 2)**

17. My teaching objectives are scientifically set and achievable
18. I am able to effectively organize online classroom teaching
19. I can ensure high-quality teacher–student interaction
20. I regularly evaluate and adjust my teaching strategies

**Sustainable development (η 3)**

21. Students’ competition scores are steadily improving
22. Students’ problem-solving abilities have significantly improved
23. Students demonstrate sustained innovative thinking
24. Students maintain long-term learning motivation

**Teacher adaptability (ξ 4)**

25. I can quickly adapt to the new teaching environment
26. I am good at innovating teaching methods and strategies
27. I am able to flexibly adjust my teaching plan
28. I actively respond to challenges in teaching

Intention for follow-up interviews: I am willing to participate in the follow-up 30–45 min in-depth interview. Contact information: The most convenient contact time: Do you have any further suggestions for this study? Thank you for your participation and support!
